# Changes in the Genotypic Characteristics of Community-Acquired Methicillin-Resistant Staphylococcus aureus Collected in 244 Medical Facilities in Japan between 2010 and 2018: a Nationwide Surveillance

**DOI:** 10.1128/spectrum.02272-21

**Published:** 2022-06-27

**Authors:** Tetsuo Yamaguchi, Itaru Nakamura, Takahiro Sato, Daisuke Ono, Ayami Sato, Shiro Sonoda, Kotaro Aoki, Yuri Miura, Shinobu Koyama, Kiyoko Tamai, Tetsuya Matsumoto, Junzo Hisatsune, Motoyuki Sugai, Yoshikazu Ishii, Kazuhiro Tateda

**Affiliations:** a Toho University, Tokyo, Japan; b Tokyo Medical University Hospital, Tokyo, Japan; c Saitama Medical Centre, Saitama, Japan; d Sakura Medical Centre, Toho University, Chiba, Japan; e Tokyo Medical and Dental University Hospital, Tokyo, Japan; f Miroku Medical Laboratory Co. Ltd., Nagano, Japan; g International University of Health and Welfare, Chiba, Japan; h National Institute of Infectious Diseases, Tokyo, Japan; Riverside University Health System Medical Center, University of California

**Keywords:** MRSA, CA-MRSA, PVL, TSST-1

## Abstract

Although community-associated methicillin-resistant Staphylococcus aureus (CA-MRSA) has emerged worldwide, no nationwide CA-MRSA surveillance has been conducted in Japan to determine the changes in its molecular characteristics over time. We aimed to characterize the molecular epidemiology of Panton-Valentine leucocidin (PVL)-positive CA-MRSA strains collected from across Japan in the past decade. We isolated 1,770 MRSA strains from the skin and pus samples of outpatients of 244 medical facilities in 31 prefectures between 2010 and 2018 (2010, 2012, 2014, 2016, and 2018). Regions, hospitals, and periods in which strains were isolated and patient age group and sex were tabulated. Staphylococcal cassette chromosome *mec* (SCC*mec*) typing, detection of virulence factor genes, and antimicrobial susceptibility testing were performed. Whole-genome analysis was performed for the PVL-positive strains isolated in 2018. All strains harbored the *mecA* gene. Compared to that in 2010, the percentage of SCC*mec* type IV increased in 2018, with a corresponding increase in the proportion of PVL-positive strains (10% to 26%). Of the isolates obtained in 2018, clonal complex 8 (CC8) was dominant among PVL-positive strains. Core-genome single-nucleotide polymorphism analysis, using whole-genome sequencing, suggested that the CC8 PVL-positive strains spread throughout Japan over the last decade. Furthermore, a unique ST22 clone carrying both the PVL- and toxic shock syndrome toxin-1-encoding genes has emerged. We demonstrated that the molecular epidemiology of CA-MRSA in Japan differs from that in Europe and the United States; thus, it is crucial to monitor the trend of changes in CA-MRSA characteristics in Japan.

**IMPORTANCE** Community-associated MRSA, which is a multidrug-resistant organism and can cause infections in otherwise-healthy individuals, has become a global problem. This paper describes a nationwide surveillance conducted in Japan to investigate changes in molecular epidemiological characteristics of CA-MRSA over the past decade and provides a detailed review of the characteristics of Panton-Valentine leucocidin (PVL)-positive strains isolated in 2018. Although CA-MRSA is rare in Japan to date, we found that the isolation of PVL-positive strains has been increasing over the past decade. In particular, the PVL-positive strains wherein CC8 was dominant exhibited high interstrain similarity, suggesting that a limited number of clones have spread over the past decade. Furthermore, a unique ST22 clone carrying both PVL-encoding and toxic shock syndrome toxin-1-encoding genes has emerged. This study shows that various changes can be observed when molecular epidemiological analysis, combined with next-generation sequencing, is conducted over a long period.

## INTRODUCTION

Methicillin-resistant Staphylococcus aureus (MRSA) was first identified in 1961 in England (1). Since the mid-1980s, the prevalence of hospital-associated MRSA (HA-MRSA) infection has increased worldwide (2). In contrast, since 2000, several countries have succeeded in reducing the prevalence of HA-MRSA that was considered to be confined to hospitals (3). In the Netherlands and Sweden, the proportion of MRSA among the detected S. aureus strains is below 5%, while in Austria, the United Kingdom, and Germany, its proportion is below 10%. However, in Italy and Portugal, the proportion of MRSA in the detected S. aureus strains is over 30%.

Since the 1990s, the emergence and increasing prevalence of community-associated MRSA (CA-MRSA) beyond health care-related environments has changed the epidemiology of MRSA (4). CA-MRSA colonizes healthy individuals and can cause skin and soft tissue infections and sometimes life-threatening necrotizing pneumonia in children and adults with no predisposing factors (5). Molecular typing studies have shown that CA-MRSA differs from HA-MRSA, as it belongs to several distinct genetic lineages and usually carries smaller staphylococcal cassette chromosome *mec* (SCC*mec*) elements and specific virulence factors, such as Panton-Valentine leukocidin (PVL), a cytotoxin (6, 7). Furthermore, CA-MRSA overexpresses toxins such as phenol-soluble modulins and hemolysins and exhibits higher virulence than HA-MRSA (8, 9).

CA-MRSA has been particularly threatening in the United States. The USA300 clone, a representative clone of CA-MRSA and a sequence type 8 (ST8) clone which carries the SCC*mec* type IVa and PVL genes, as well as the arginine catabolic mobile element (ACME), a mobile genetic element, had spread throughout the United States by 2004 (10). According to reports by the Centers for Disease Control and Prevention (CDC) on invasive S. aureus disease, incidences of hospital-onset (HO) and health care-associated community-onset (HACO) MRSA have been on the decline since 2004, while the incidence of CA-MRSA has only mildly declined, from 5.6/100,000 population in 2005 to 4.8/100,000 in 2017 (11, 12). The USA300 clone is considered a highly pathogenic clone, and many severe infection cases have been reported. Outside the United States, the Southwest Pacific clone (ST30/SCC*mec* IVc/PVL^+^) around the world, the Taiwan clone (ST59/SCC*mec* V/PVL^+^) in South Asia, and the European clone (ST80/SCC*mec* IVc/PVL^+^) in Europe are the dominant clones (5, 13). However, because there are reports of increased detection of the USA300 clone in Europe, its global spread is feared (13–15).

Japan is known to be a country where MRSA is endemic; in 2000, about 70% of the S. aureus strains detected in Japanese health care facilities were MRSA, but in 2014, the MRSA rate fell to less than 50% (https://janis.mhlw.go.jp/english/index.asp). However, this was only in relation to health care-associated infections and not community-associated ones. Several studies have suggested that PVL-positive CA-MRSA is rare in the Japanese population; however, the strains mentioned in these reports also included HA-MRSA or were only isolated from a few study centers (16–18). Therefore, based on these reports, it is difficult to understand the epidemiology of CA-MRSA clones in Japan. We conducted a surveillance of CA-MRSA in 2010 and 2012 (19). Only 10.4% and 8.3% of the CA-MRSA strains from 2010 and 2012 were PVL positive, respectively, and only 0.8% and 2.1% were the USA300 clone in 2010 and 2012, respectively, suggesting that the prevalence of highly pathogenic CA-MRSA was low in Japan at that time.

We continued our nationwide surveillance and collected a total of 1,770 CA-MRSA strains from 2010 to 2018. Here, we report the molecular and epidemiological characteristics of CA-MRSA strains isolated from patients in a large number of health care facilities throughout Japan. This study is the first longitudinal analysis of epidemiological data of CA-MRSA for about 10 years in Japan, and it includes many new findings.

## RESULTS

### Clinical and epidemiological data.

In total, 46,549 skin and pus samples obtained from outpatients were sent to the Miroku Medical Laboratory Co. (MML) for culture during the study period, and pathogens were detected in 29,813 samples (Table 1). Pathogens obtained from these samples included 9,726 S. aureus isolates (32.6%), among which 1,928 (19.8%) were MRSA. When MRSA was isolated repeatedly from the same patient, this was considered one strain. A total of 1,770 MRSA strains isolated from the skin and pus samples collected from 244 medical facilities in 31 prefectures of Japan were sent to Toho University for molecular characterization. We isolated MRSA strains from 78, 102, 94, 102, and 100 facilities in 2010, 2012, 2014, 2016, and 2018, respectively. Furthermore, MRSA was detected in only 1 year during the study period in 124 facilities, and 180 strains were isolated from these facilities. The remaining 1,590 strains were isolated from 120 facilities where MRSA was isolated in more than 1 year. Most MRSA samples (46.8%) were from the Kanto area, which has the highest population density, followed by the Chubu area (38.0%), where MML is located. Among the patients from which CA-MRSA strains were isolated, 31.6% were under 10 years of age, and the ratio of males to females was 52.9% to 46.0% (unknown, 1.1%).

**TABLE 1:** Details of bacterial strains isolated in this study by year

Sample category	2010^*a*^	2012^*a*^	2014	2016	2018
All samples for culture	134,282	213,524	205,412	215,460	182,674
All outpatient samples for culture	64,865	103,822	102,522	112,951	94,655
All samples from skin or pus of outpatients	5,577	9,465	10,670	10,794	10,043
Culture-positive samples	3,957	6,428	6,831	6,741	5,856
S. aureus isolates (% of all positive cultures)	1,436 (36.3)	2,145 (33.4)	2,390 (35.0)	2,092 (31.0)	1,663 (28.4)
MRSA isolates (% of all S. aureus isolates)	260 (18.1)	413 (19.3)	463 (19.4)	420 (20.1)	372 (22.4)
Analyzed MRSA strains	241	384	401	397	347
Samples with virulence gene for:					
PVL (% of all MRSA strains)	25 (10.4)	32 (8.3)	53 (13.2)	81 (20.4)	92 (26.5)
TSST-1 (% of all MRSA strains)	81 (33.6)	114 (29.7)	122 (30.4)	89 (22.4)	75 (21.6)
PVL and TSST-1 (% of all MRSA strains)		1 (0.3)			5 (1.4)

a Data include the reported data of the isolates in 2010 and 2012 (19); the data on the isolates obtained from 2014, 2016, and 2018 were added for comparison.

### SCC*mec* typing and virulence gene analysis of MRSA.

**FIG 1:**
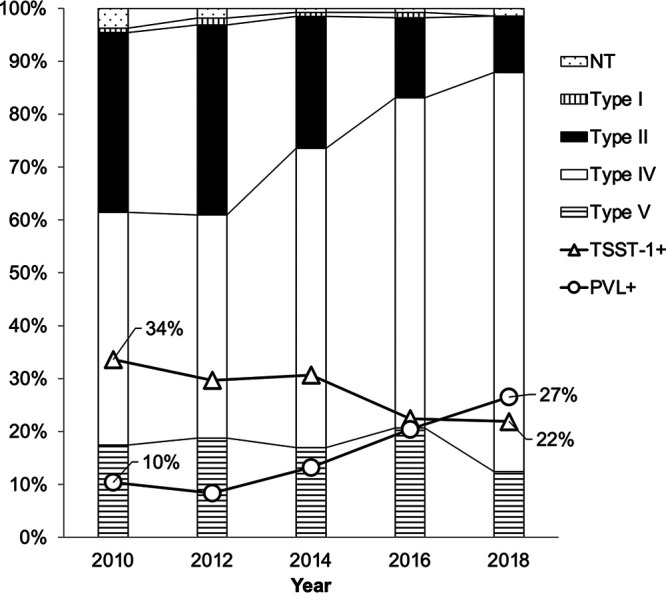
SCC*mec* typing of CA-MRSA in Japan. Data regarding the isolates collected from 2010 and 2012 have been reported previously (19); data on the isolates obtained from 2014, 2016, and 2018 were added for comparison. SCC*mec*, staphylococcal cassette chromosome *mec*; NT, nontypeable SCC*mec*; type I, SCC*mec* type I; type II, SCC*mec* type II; type IV, SCC*mec* type IV; type V, SCC*mec* type V; TSST-1+, toxic shock syndrome toxin-1 gene-positive strains; PVL+, Panton-Valentine leucocidin gene-positive strains.

**FIG 2:**
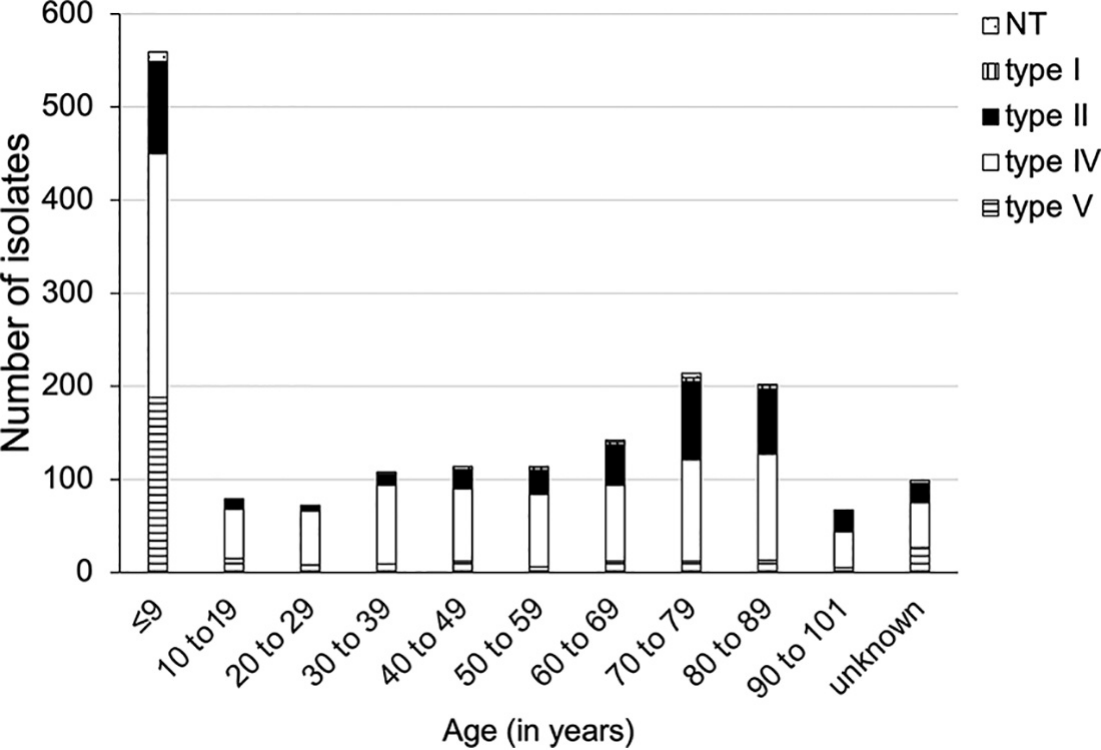
Age distribution of isolates for each SCC*mec* type (from 2010 to 2018). SCC*mec* types IV and V were mostly isolated from patients ≤9 years of age (26.0% and 61.2%, respectively). NT, nontypeable SCC*mec*; SCC*mec*, staphylococcal cassette chromosome *mec*.

We detected *mecA* in all the strains. The prevalence of SCC*mec* type IV increased from 44.0% to 75.8% between 2010 and 2018 (Fig. 1). Patients with SCC*mec* type IV strains were significantly younger than those with type II strains, and patients with type V strains were significantly younger than those with type IV (Fig. 2). Furthermore, SCC*mec* type II strains were frequently isolated from patients under 10 (23.8%) and over 60 years of age (52.9%).

Results of the virulence gene analysis are shown in Table 1. The PVL-positive rate among the MRSA strains increased by approximately 3-fold, from 10.4% in 2010 to 26.5% in 2018. The prevalence of toxic shock syndrome toxin 1 (TSST-1)-positive strains decreased from 33.6% in 2010 to 21.6% in 2018. Only one strain (0.3%) carrying both the PVL and TSST-1 genes was isolated in 2012, but five such strains (1.4%) were isolated in 2018 alone.

### Antimicrobial susceptibilities.

The MIC_50_ and MIC_90_ of each SCC*mec* type for each agent are shown in Table 2. Univariate analysis of the relationship between each SCC*mec* type and antimicrobial MICs showed that there were significant differences (*P* < 0.05) in the relationships between SCC*mec* types II, IV, and V and the following antimicrobial agents: cefazolin (CFZ), cefmetazole (CMZ), flomoxef (FMOX), imipenem (IPM), and levofloxacin (LVX). For CFZ, CMZ, FMOX, and LVX, the MIC_50_ for SCC*mec* type IV strains was lower than that for type II strains, and the MIC_50_ for type V strains was lower than that for type IV strains. In addition, for clindamycin (CLI), minocycline (MIN), and IPM, the MIC_50_ for SCC*mec* type II strains was higher than that for type IV and V strains. For gentamicin (GEN) and erythromycin (ERY), the MIC_50_ was high for all SCC*mec* type strains; for sulfamethoxazole-trimethoprim (SXT) and vancomycin (VAN), the MIC_50_ was low for all SCC*mec* type strains.

**TABLE 2:** MIC_50_ and MIC_90_ levels of the 12 antibiotics tested on the MRSA strains isolated in 2010, 2014, and 2018, based on SCC*mec* type^*a*^

Antibiotic group and agent (MIC range		MIC_50_, MIC_90_ (μg/mL) for SCC*mec* type			
		All types	II (*n* = 218)	IV (*n* = 596)	V (*n* = 153)
Non-beta-lactams					
Gentamicin (≤0.25 to >8 μg/mL)		>8, >8	>8, >8	>8, >8	>8, >8
Levofloxacin (≤0.25 to >4 μg/mL)		4, >4	>4, >4	4, >4	≤0.25, 0.5
Clindamycin (≤0.06 to >2 μg/mL)		0.25, >2	>2, >2	0.12, >2	0.25, >2
Erythromycin (≤0.12 to >4 μg/mL)		>4, >4	>4, >4	>4, >4	>4, >4
Minocycline (≤2 to >8 μg/mL)		≤2, >8	8, >8	≤2, 8	≤2, ≤2
Sulfamethoxazole-trimethoprim (≤9.5/0.5 to >38/2 μg/mL)		≤9.5/0.5, ≤9.5/0.5	≤9.5/0.5, ≤9.5/0.5	≤9.5/0.5, ≤9.5/0.5	≤9.5/0.5, ≤9.5/0.5
Vancomycin (≤0.5 to 4 μg/mL)		1, 1	1, 1	1, 1	1, 1
Beta-lactams^*b*^					
Cefazolin (≤0.5 to >16 μg/mL)		8, >16	>16, >16	8, >16	1, 2
Cefmetazole (≤2 to >32 μg/mL)		8, 32	32, >32	8, 16	4, 8
Flomoxef (≤0.5 to >16 μg/mL)		4, >16	>16, >16	4, 8	2, 4
Imipenem (≤0.25 to >8 μg/mL)		≤0.25, >8	>8, >8	≤0.25, 1	≤0.25, 0.25

a Data include the reported MIC values of the isolates of 2010 (19).

b MICs of beta-lactams were low for some MRSA strains. However, even if the MIC of the antibiotic is low, the antibiotic may not be clinically effective.

**FIG 3:**
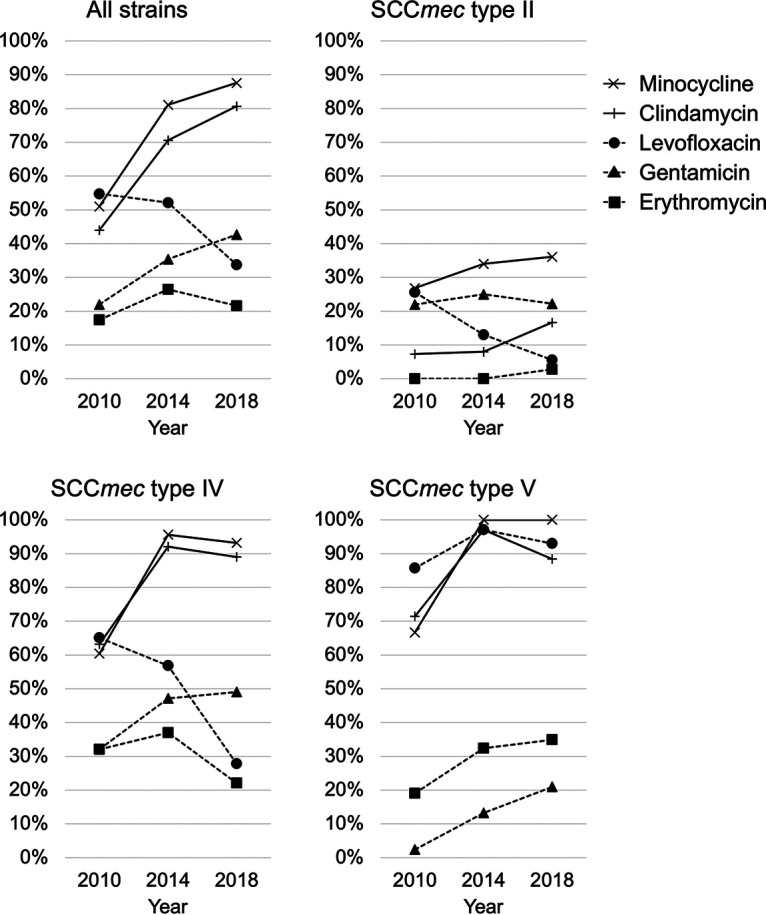
Trends in the percentage of strains susceptible to non-beta-lactam antibiotics over the study period. Data regarding the isolates collected from 2010 have been reported previously (19). Data on the isolates obtained from 2014 and 2018 were added for comparison.

When comparing strain susceptibility according to the detection year, the percentage of strains susceptible to MIN and CLI increased in 2018 (87.6% and 80.7%, respectively) compared to levels in 2014 (81.0% and 70.6%, respectively) and in 2010 (51.0% and 44.0%, respectively) (Fig. 3). The increased percentage of the SCC*mec* type IV and type V strains susceptible to MIN and CLI in 2014, compared to 2010, contributes to the increase in the susceptibility of all strains. Susceptibility to levofloxacin decreased over time, with 54.8%, 52.1%, and 33.7% of strains being susceptible in 2010, 2014, and 2018, respectively. SCC*mec* type II and type IV strains, but not SCC*mec* type V strains, are becoming quinolone resistant.

### Whole-genome sequencing and analysis of PVL-positive CA-MRSA.

Among the 92 PVL-positive strains detected in 2018, CC8 strains were identified most frequently (59 isolates), followed by CC22 (15 isolates), and four additional CCs, namely, CC5 (1 isolate), CC30 (8 isolates), CC59 (7 isolates), and CC398 (1 isolate) (Table 3; see also Table S1 in the supplemental material). Most CC8 had the same genetic profile as that of USA300_FPR3757: the SCC*mec* type was IVa and carried the *sek*, *seq*, *hglA*, *hlgB*, *hlgC*, *lukE*, *lukD*, *scn*, *sak*, and ACME genes. Only one strain (CAM-1934) had the same genetic profile as that of USA300-LV: the SCC*mec* type was IVc and it did not carry ACME genes. The next most frequently detected lineage was CC22, which had the same sequence type as that of UK-EMRSA-15 (i.e., ST22/SCC*mec* type IV and PVL genes absent), known as an HA-MRSA clone. Among the 15 isolates, 10 strains were subtype IVc and the remaining 5 were subtype IVa. These ST22/PVL-positive strains carried various enterotoxins; in particular, the ST22/SCC*mec* IVa clone carried *sec*, *sel*, and *tst-1*, in addition to *seg*, *sei*, *sem*, *sen*, *seo*, and *seu*.

**TABLE 3:** Molecular characteristics of PVL-positive MRSA isolates obtained in 2018

Genotype					Virulence genes for:^*a*^			Resistance genes^*a*^	
CC	MLST	Allelic profile	No. of isolates (*n* = 92)	SCC*mec* type	Enterotoxins	Pore-forming toxins	Other proteins	Acquired	Mutations
CC5 (*n* = 1)	ST5	5-1-4-1-4-12-1-10	1	IVc	*seg, sei, sem, sen, seo, seu*	*hlgA, hlgB, hlgC, lukE, lukD*	*scn, sak*	*dfrG*	
CC8 (*n* = 59)	ST8	3-3-1-1-4-4-3	55	IVa	*sek, seq*	*hlgA, hlgB, hlgC, lukE, lukD*	*scn, sak,* ACME	*msr*(A)	*gyrA* (p.S84L), *grlA* (p.S80Y)
			1	IVc	*sek, seq*	*hlgA, hlgB, hlgC, lukE, lukD*	*scn, sak*		
	ST6585^*b*^	3-3-1-1-801^*c*^-4-3	1	IVa	*sek, seq*	*hlgA, hlgB, hlgC, lukE, lukD*	*scn, sak,* ACME	*aph(3′)-III, msr*(A)*, mph*(C)	*gyrA* (p.S84L), *grlA* (p.S80Y)
	ST6582^*b*^	3-3-1-1-4-742-3	1	IVa	*sek, seq*	*hlgA, hlgB, hlgC, lukE, lukD*	*scn, sak,* ACME	*aph(3′)-III, aac(6′)-aph(2″), msr*(A)*, mph*(C)	gyrA (p.S84L), *grlA* (p.S80Y)
	ST6562	3-3-1-1-4-739-3	1	IVa		*hlgA, hlgB, hlgC, lukE, lukD*	*scn, sak,* ACME	*aph(3′)-III, msr*(A)*, mph*(C)	*gyrA* (p.S84L), *grlA* (p.S80Y)
CC22 (*n* = 15)	ST22	7-6-1-5-8-8-6	10	IVc	*seg, sei, sem, sen, seo, seu*	*hlgA, hlgB, hlgC*	*scn, sak*	*aac(6′)-aph(2″), erm*(C)	*gyrA* (p.S84L), *grlA* (p.S80F)
			5	IVa	*seg, sei, sem, sen, seo, seu, sec, tst-1, sel*	*hlgA, hlgB, hlgC*	*scn, sak*	*aac(6′)-aph(2″)*	*gyrA* (p.S84L), *grlA* (p.S80F)
CC30 (*n* = 8)	ST30	2-2-2-2-6-3-2	7	IVc	*seg, sei, sem, sen, seo, seu*	*hlgA, hlgB, hlgC*	*scn, sak*		
	ST6586^*b*^	2-2-2-2-6-746^*c*^-2	1	IVc	*seg, sei, sem, sen, seo, seu*	*hlgA, hlgB, hlgC*	*scn, sak*		
CC59 (*n* = 7)	ST59	19-23-15-2-19-20-15	1	IVa		*hlgA, hlgB, hlgC*	*scn*	*aph(3′)-III, erm*(B)	
			6	V	*sek, seq, seb*	*hlgA, hlgB, hlgC*	*scn*		
CC398 (*n* = 1)	ST1232	3-35-167-2-20-26-39	1	V		*hlgA, hlgB, hlgC*	*scn*	*erm*(A), *tet*(K), *fosB4*	
NT	ST338	19-23-15-48-19-20-15	1	V		*hlgA, hlgB, hlgC*	*scn*	*aph(3′)-III, erm*(B)	

a Genes found in ≥80% of the strains.

b New sequence type in this study.

c New allele in this study.

### Core-genome single-nucleotide polymorphism analysis of the PVL-positive strains.

**FIG 4:**
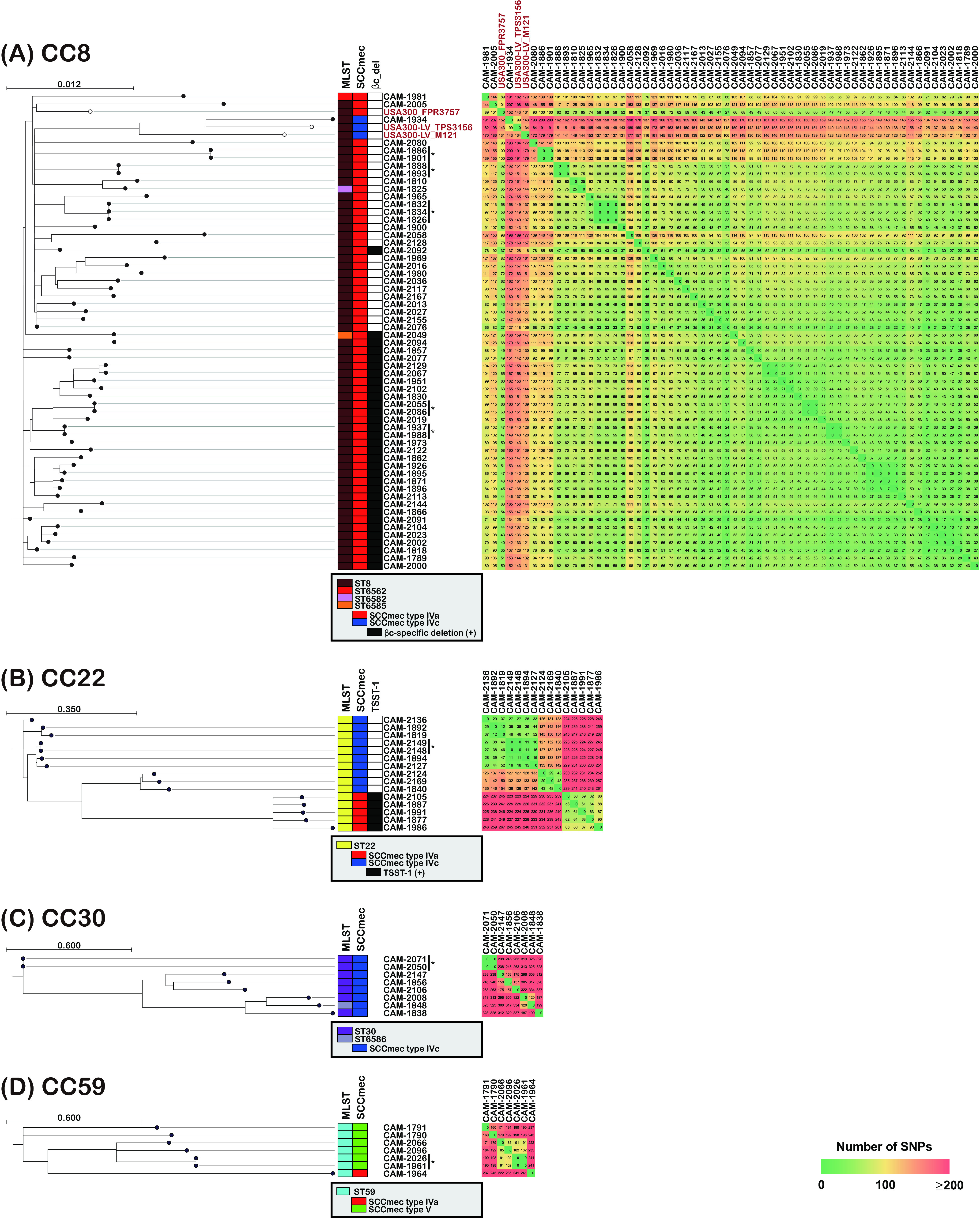
Phylogenetic trees of the PVL-positive strains in 2018. (A) Sixty-two strains of MRSA CC8. USA300_FPR3757 and two USA300 Latin America variant (USA300-LV) strains (USA300-LV_M121 and USA300-LV_TPS3156), a USA300 clonal subtype frequently reported in Latin America, were included as reference strains (reference strains are indicated using white circles and red text). A core genome region, amounting to 85.7% (2,462,130/2,872,769 bp), was shared with the genome of the reference strain S. aureus USA300_FPR3757 (ST8). An explanation of the βc-specific deletion is provided in Materials and Methods. (B) Fifteen strains of MRSA CC22. A core genome region, amounting to 91.5% (2,604,425/2,846,320 bp), was shared with the genome of the reference strain S. aureus UK-EMRSA-15 (ST22). (C) Eight strains of MRSA CC30. A core genome region, amounting to 95.4% (2,651,817/2,778,854 bp), was shared with the genome of the reference strain S. aureus ATCC 25923 (ST30). (D) Eight strains of MRSA CC59. A core genome region, amounting to 95.7% (2,669,274/2,788,636 bp), was shared with the genome of the reference strain S. aureus M013 (ST59). *, there were no SNPs.

Most CC8 strains showed high similarity to USA300_FPR3757 (Fig. 4A). Of the 59 strains, 58 were SCC*mec* type IVa. Moreover, the number of single-nucleotide polymorphisms (SNPs) between USA300_FPR3757 and each strain was less than 101. In particular, the 32 strains with the βc-specific deletion formed a very similar group with fewer than 80 SNPs; these strains are thought to have diverged from a common original strain and have been spreading in Japan over the past decade. Only one strain, CAM-1934, was SCC*mec* type IVc, and this strain had the lowest similarity to USA300_FPR3757. This strain was thought to have diverged from USA300-LV, a variant of the USA300 clone.

CC22 was divided into two major genotypes, namely, ST22/SCC*mec* IVa and ST22/SCC*mec* IVc (Fig. 4B). Furthermore, all five strains of the ST22/SCC*mec* IVa clone harbored *lukS-pv*, *lukF-pv*, and *tst-1* genes. CC30 showed a relatively low interstrain similarity, with only two strains having no SNPs, while the other strains had more than 100 SNPs (Fig. 4C). This was also observed in CC59, which was similar to CC30 (Fig. 4D).

## DISCUSSION

In this study, we analyzed the changes in the molecular and epidemiological characteristics of CA-MRSA in Japan during 2010 to 2018. Our results showed that the ratio of MRSA to all S. aureus strains in community-acquired infections was approximately 20% and was lower than that observed in hospitalized patients in Japan (47.5% in 2018) (https://janis.mhlw.go.jp/english/index.asp). However, the ratio of MRSA to all S. aureus strains increased slightly from 18.1% to 22.4% between 2010 and 2018, and the percentage of PVL-positive strains among CA-MRSA isolates increased from 10.4% in 2010 to 26.5% in 2018.

Results of this study reiterate that the molecular epidemiology of MRSA strains in Japan differs from that of strains in Europe and the United States. In Japan, the isolation frequency of PVL-positive CA-MRSA strains is low, and CA-MRSA/J (ST8/SCC*mec* IV/TSST-1^+^), which is unique to Japan, is often isolated (20). In addition, ST89/SCC*mec* IV/*eta* or *etb*-positive strains have been isolated in impetigo, and in recent years, ST1/SCC*mec* IV/*cna*-positive strains have been isolated (21, 22). In this study, TSST-1-producing CA-MRSA strains were isolated most frequently in 2010; however, in 2018, the final year of this study, PVL-positive strains were more prevalent than TSST-1-positive strains. These results suggest that significant changes occurred in the molecular epidemiology of CA-MRSA in Japan during the period covered by this study.

For PVL-positive strains, which have increased over the decade, we determined multilocus sequence types (MLSTs) of isolates in 2010 and in 2018, thus allowing comparisons. In the 2010, 25 PVL-positive strains (10.4% of MRSA) were isolated, with the ST30/SCC*mec* IVc clone being the most frequently isolated, with 12 strains (5.0% of MRSA). The second most common clone was the ST8/SCC*mec* IVc clone, with seven isolates (2.8% of MRSA), followed by the ST8/SCC*mec* IVa clone (also known as the USA300 clone), with two isolates (0.8% of MRSA). Only one (0.4%) of each of the other clones (ST22/SCC*mec* IVc, ST452/SCC*mec* IVc, ST59/SCC*mec* V, ST154/SCC*mec* IVn) was detected. In 2018, 92 PVL-producing strains were isolated, and CC30, including ST30/SCC*mec* IVc, the most frequently isolated clone in 2010, was identified in only eight isolates (2.3% of all MRSA analyzed). Furthermore, ST8/SCC*mec* IVc, the second most frequently isolated strain in 2010, was identified in only one isolate in 2018. In contrast, although only two strains of ST8/SCC*mec* IVa/PVL^+^/ACME^+^, which has the same genomic type as the USA300 clone, were detected in Japan in 2010, this number increased remarkably in 2018 to 58 strains (16.7% of MRSA in 2018). Even in the United States, where the USA300 clone is now widespread, the clone was not detected before 2000; however, in 2004, it accounted for the majority of the CA-MRSA cases and subsequently became the most common MRSA clone detected in health care facilities in the United States (10, 23). In Japan, if the current trend of the increasing rates of PVL-positive CA-MRSA strains continues, PVL-positive CA-MRSA strains may become the dominant clone.

According to whole-genome sequencing (WGS) via next-generation sequencing (NGS), CC8 was the most frequently detected PVL-positive lineage in 2018. SNP analysis revealed that 58 of the 59 CC8 strains showed fewer than 101 SNPs compared to USA300_FPR3757. This result suggests that these strains have spread rapidly in Japan over the last decade. Next, many strains of the ST22 clone, including the double-positive PVL^+^ and TSST-1^+^ ST22 clone (ST22-PT clone), were isolated in 2018. The most famous ST22 clone is EMRSA-15 (ST22/SCC*mec* IVh), also known as HA-MRSA; however, strains with a different genetic background from that of this clone have been isolated as CA-MRSA (24). Recently, the “Gaza clone” (ST22/SCC*mec* IVa/TSST-1^+^) has been reported to be CA-MRSA (25, 26). One of the characteristics of this strain is that its SCC*mec* type is IVa, unlike EMRSA-15, and it often possesses the TSST-1 gene. Gaza clones that possess both the TSST-1 and PVL genes have been reported, and the ST22-PT clone isolated in this study was genetically close to the Gaza clone. Furthermore, we observed an intrafamilial transmission case involving the ST22-PT clone, which causes fatal necrotizing pneumonia and sepsis, suggesting that the ST22-PT clone is highly virulent (27). The five ST22-PT strains detected in our study formed a group and harbored fewer than 100 SNPs. However, these five strains were detected in different hospitals, thus ruling out the possibility of an outbreak within a single medical institution and suggesting that this specific PT-CA-MRSA clone is spreading in Japan. Furthermore, a PVL-positive ST1232 (CC398) clone was detected; CC398 is a known livestock-associated MRSA but is sometimes detected as CA-MRSA and includes the PVL-positive strains (28, 29). A PVL-positive ST1232 clone has been isolated in Japan in the past (30).

The results of this study show that among MRSA strains, several strains have low β-lactam MICs; furthermore, β-lactam MICs for SCC*mec* type IV and type V were lower than those for SCC*mec* type II. Because such strains harbor *mecA*, they are likely to become resistant even if β-lactam MICs are low, and thus, β-lactams should not be used. However, in recent years, the strategies of utilizing β-lactams against MRSA have changed, including their incorporation into combination therapy (31–34). The susceptibility of MRSA to β-lactams may be important in the selection of therapeutic agents in the future. Excluding β-lactams, the MIC_50_ for SCC*mec* type II strains was higher than that for type IV and V strains for LVX, CLI, and MIN. Furthermore, compared to that in 2010, the percentage of strains susceptible to MIN and CLI increased and the percentage of strains susceptible to LVX decreased in 2018. The percentage of strains susceptible to GEN and ERY was low, while the percentage of strains susceptible to SXT was high. These results suggest that MIN, CLI, and SXT are potential therapeutic options when skin and soft tissue infections caused by MRSA are suspected.

This study had several limitations. First, since it was conducted in collaboration with a laboratory with specific constraints related to patient data availability, the clinical information of the enrolled patients was limited. Thus, further studies are required to assess whether the strain characteristics are linked to the infection type and severity in patients. Second, the study was adjusted to include more CA-MRSA strains by targeting outpatients and limiting the samples from skin origin. However, it is possible that patients with a clinical background of HA-MRSA were included in the study. Third, MRSA strains were collected from outpatients of all age groups but were mostly isolated from patients under 10 years of age. We believe that this indicates that skin and soft tissue infections caused by S. aureus, especially CA-MRSA, are common in children. However, in the current study, it was not possible to tabulate the age distribution of the entire patient population whose samples were sent for culture. It remains unclear whether the age of the entire patient population for whom samples were sent for culture was low or the age of the patient population from whose samples CA-MRSA was isolated was lower. Fourth, in this study, only PVL-positive MRSA strains were analyzed by NGS; a broader analysis is needed to understand the lineages of PVL-negative MRSA.

In summary, the number of CA-MRSA strains, including the PVL-positive strains isolated from skin and soft tissue infections, has gradually increased since 2010. To the best of our knowledge, this study is the first to report and describe CA-MRSA isolates from Japan collected over the past decade and to indicate that the molecular epidemiology of CA-MRSA in Japan is unique and different from that in Europe and the United States. Taken together, our study highlights the need to continuously monitor the trends in CA-MRSA prevalence and devise novel strategies for the management and control of infections in Japan.

## MATERIALS AND METHODS

### Bacterial strains and definitions of CA-MRSA.

We collaborated with the Miroku Medical Laboratory Co. (Nagano, Japan), a contract-based microbiology laboratory that analyzes clinical samples for over 200 medical facilities across Japan. We isolated S. aureus from skin and pus samples collected from outpatients registered in 2010 (February to September), 2012 (February 2012 to January 2013), 2014 (January to December), 2016 (January to December), and 2018 (January to December). These S. aureus strains were tested for antimicrobial susceptibility to oxacillin (OXA), using the broth microdilution method, and to cefoxitin (FOX), using the disc diffusion method according to the Clinical and Laboratory Standard Institute (CLSI) reference methods (35). The strains with OXA MICs of ≥4 µg/mL or zone diameters for FOX of ≤21 mm were identified as MRSA. Since this study was limited to outpatients, there was a possibility that these cases of skin infection were community-onset infections. Therefore, we treated the MRSA isolates from the skin samples of outpatients as CA-MRSA. These CA-MRSA strains were sent to our laboratory for molecular characterization. Data regarding the isolates collected from 2010 and 2012 have been reported previously (19); the data on the isolates obtained from 2014, 2016, and 2018 were added for comparison and review of the molecular epidemiological changes over the past decade. Due to the restricted contract between MML and the medical facilities, we could collect only limited clinical data from the patients, including the date of isolation, region, sex, and age group. The samples were collected by MML and anonymized so that no individuals were identifiable. Moreover, MML provided the patients with information on the opportunity to refuse to participate in the study (opt-out) on its website. The research protocols were approved by the Safety Committee for Pathogens of Toho University (20-53-101) and the Ethics Committee of the Faculty of Medicine, Toho University (A20013_A17019). Since some of the SCC*mec* types are rare in Japan, the following strains were used as positive controls in this study: NCTC10442 (SCC*mec* type I), N315 (SCC*mec* type IIa), JCSC3063 (SCC*mec* type IIb), 85/2082 (SCC*mec* type III), JCSC4744 (SCC*mec* type IVa), JCSC2172 (SCC*mec* type IVb), JCSC4788 (SCC*mec* type IVc), JCSC4469 (SCC*mec* type IVd), JCSC4796 (SCC*mec* type IVg), WIS (SCC*mec* type V), and JCSC6774 (USA300 clone). These strains were provided by Keiichi Hiramatsu (Juntendo University, Tokyo, Japan).

### Antimicrobial susceptibility testing.

To compare changes in antimicrobial susceptibility over the past decade, MRSA strains isolated during 2010, 2014, and 2018 (241, 401, and 347 strains, respectively) were selected. The MICs for each isolate were determined using a broth microdilution assay according to the CLSI reference methods (35).

The antimicrobial susceptibility of each strain was tested using ready-made dry plates (DP32; Eiken Chemical Co. Ltd., Tokyo, Japan) containing 13 antimicrobial agents, including OXA, FOX, CFZ, CMZ, FMOX, IPM, GEN, MIN, ERY, CLI, SXT, LVX, and VAN. The MIC values for the susceptibility criteria were determined according to CLSI document M100-Ed30 (36), and those for beta-lactams were determined according to CLSI document M100-S22 (37). The Staphylococcus aureus ATCC 29213 strain was used for quality control of the broth microdilution assay. The MICs for the isolates collected in 2010 have been reported previously (19); the data on the isolates obtained from 2014 and 2018 were added for comparison over the past decade.

### SCC*mec* typing and virulence gene analysis.

The SCC*mec* elements are currently classified based on the combination of two essential components, the *mec* gene complex and the *ccr* gene complex (38, 39). We used the basic PCR strategy established by Ito et al. (39, 40) to classify *ccr* gene complexes into type 1 to type 5 by using eight different primers (primers used for typing were α1 and βc for type 1, α2 and βc for type 2, α3 and βc for type 3, α4.2 and β4.2 for type 4, and γR and γF for type 5). However, some type 2 *ccr* gene complexes that cannot be detected using this method have been reported (41). It has been shown that this is due to a specific 11-bp deletion in the βc binding site of the type 2 *ccr* gene complex. This βc-specific deletion is relatively common in CA-MRSA clones in Japan, especially in the USA300 clone. Therefore, we designed a new primer sequence which was different at the βc region (primer β2′, 5′-TGGACTTGGGGTTTTTGA-3′) and could bind to the type 2 *ccr* gene complex even in the presence of a βc-specific deletion (see Fig. S1 in the supplemental material for details). In this study, in cases where the *ccr* gene complex was not detected using the standard method, an additional PCR assay with the primer pair α2 and β2′ was performed to detect the type 2 *ccr* gene complex. If the PCR product was confirmed by this PCR, the strain was classified as *ccr* type 2.

Staphylococcal virulence genes were detected using a PCR assay and previously reported primers (42–46). The target genes included the PVL gene (*lukSF-pv*) and TSST-1 gene (*tst-1*).

### Genome sequencing of PVL-positive strains.

We performed draft WGS of PVL-positive strains from the samples isolated in 2018 to characterize the highly virulent clones. Genomic DNA was extracted using the QIAamp DNA minikit (Qiagen, Hilden, Germany). DNA libraries were prepared using the Enzymatics 5X WGS reagents (BioStream Co., Ltd., Tokyo, Japan) and pooled. Sequencing was performed using the Illumina HiSeq X FIVE platform (Illumina, Inc.) at Macrogen Japan Corporation (Tokyo, Japan). The Illumina reads were assembled using the CLC Genomics Workbench software ver. 20.0.4 (Qiagen), and the assembled contigs were analyzed using ResFinder 3.2 to identify the drug resistance genes, with MLST 1.8 for multilocus sequence typing and SCC*mec* Finder 1.2 for SCC*mec* typing (all available on the Centre for Genomic Epidemiology website, http://www.genomicepidemiology.org/services/) (47–49). For drug resistance- and toxin-encoding genes, 90% similarity and 60% reference sequence length were considered positive results.

### Core-genome SNP-based phylogenetic analysis of PVL-positive strains.

Core-genome SNP-based phylogenetic analysis using Illumina sequencing data was performed for the PVL-positive strains obtained in 2018, which belonged to the four largest clonal complexes (CC8, CC22, CC30, and CC59), comprising more than seven strains. The Illumina reads were aligned to the genomic sequence of the reference strain using the Burrows-Wheeler Aligner with the SW algorithm (50). To ensure high similarity, different gene sequences were selected as reference sequences for each CC; the reads of CC8, CC22, CC30, and CC59 strains were mapped to USA300_FPR3757 (accession number NC_007793), UK-EMRSA-15 (accession number NZ_CP007659), ATCC 25923 (accession number NZ_CP009361), and M013 (accession number: CP003166), respectively. Core-genome regions were extracted using the Sequence Alignment/Map software (SAMtools mpileup, version 1.1) (51) and read using VarScan (version 2.3.7) mpileup2cns (52). Maximum-likelihood phylogenetic trees were constructed using PhyML (53). Using these maximum-likelihood trees as the initial trees, we estimated homologous recombination events in which DNA fragments from beyond the phylogenetic clade were imported, and we constructed a clonal phylogeny with corrected branch lengths using ClonalFrameML (54). The core genome, excluding the homologous recombination sequences estimated using ClonalFrameML, was analyzed for SNP detection.

### Statistical analysis.

We performed statistical analysis using SPSS software, version 27 (IBM, Chicago, IL, USA), with a two-sided significance level of 5% for all statistical tests. To investigate associations between each SCC*mec* type and antimicrobial susceptibility or patient age distribution, we applied the Kruskal-Wallis test, followed by the Dann-Bonferroni test, for multiple comparisons. To compare antimicrobial susceptibility to each SCC*mec* statistically, the MIC values of antibiotics for each strain were used. If the MIC of each antibiotic exceeded the maximum concentration of the antibiotics measured, then twice the maximum concentration value was considered the MIC for statistical analysis; if the MIC was less than the minimum concentration measured, the minimum concentration was considered the MIC for statistical analysis. (For example, if the MIC was >128 μg/mL, 256 μg/mL was considered the MIC, whereas if the MIC was ≤32 μg/mL, 32 μg/mL was considered the MIC).

### Data availability.

Table S1 in the supplemental material shows the type and accessory genes of 92 PVL-positive MRSA isolates obtained in 2018, along with their run accession numbers. These read data for the 92 PVL-positive strains have been deposited in the DNA Data Bank of Japan (DDBJ) under BioProject accession number PRJDB11170.
