# Supplementary material for: Changes in the Genotypic Characteristics of Community-Acquired Methicillin-Resistant Staphylococcus aureus Collected in 244 Medical Facilities in Japan between 2010 and 2018: a Nationwide Surveillance
Source: Microbiol Spectr. 2022 Jun 27;10(4):e02272-21. doi: 10.1128/spectrum.02272-21 (PMC9431082; doi:10.1128/spectrum.02272-21)
Supplement: Supplemental file 1 — Supplemental material. Download spectrum.02272-21-s0001.pdf, PDF file, 0.1 MB [file spectrum.02272-21-s0001.pdf]

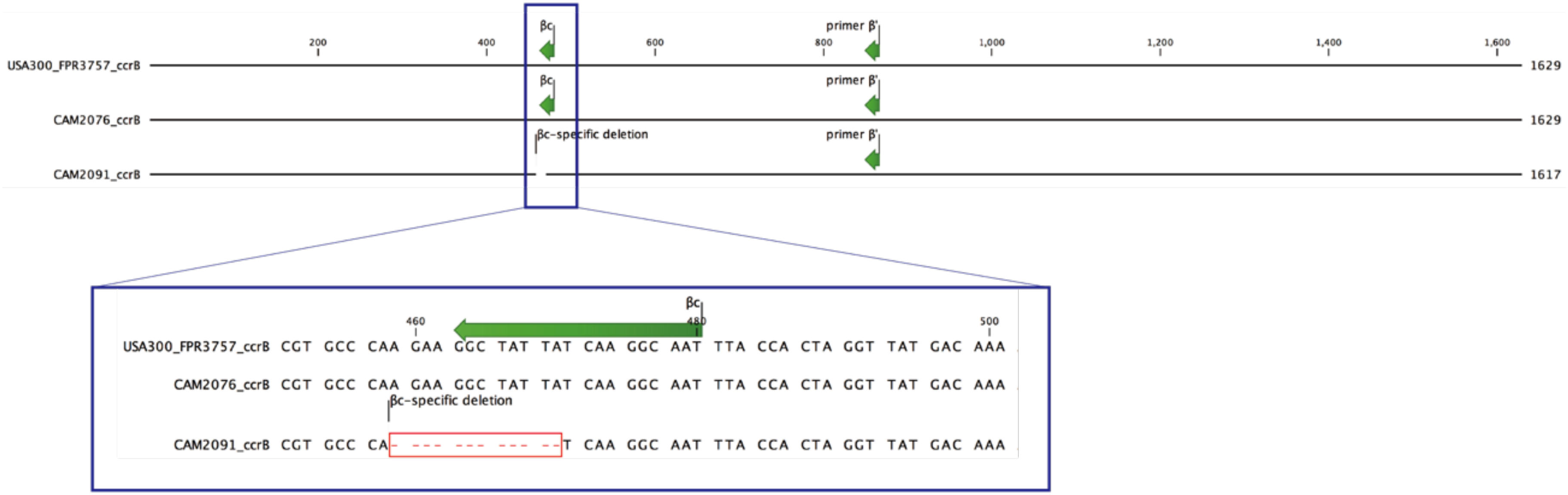

Supplementary Figure S1. The βc-specific deletion and a new primer β2′ sequence. Some type 2 *ccr* gene complexes, such as CAM2091, cannot be detected using primer βc due to specific 11-bp deletions in the βc binding site. Therefore, we designed a new primer sequence that differed at the βc region (primer β2′: TGGACTTGGGGTTTTTGA) and could bind to the type 2 *ccr* gene complex despite a βc-specific deletion. USA300_FPR3757 and CAM2076 possess complete type 2 *ccr* gene complexes. CAM2091 possesses the incomplete type 2 *ccr* gene complex with a βc-specific 11-bp deletion.
